# COVID-19 Vaccination in Home Health and Hospice: Barriers to Vaccination and Results From a Home Vaccination Program

**DOI:** 10.1093/geroni/igab046.564

**Published:** 2021-12-17

**Authors:** Robert Rosati, Steven Landers, Tami Videon

**Affiliations:** VNA Health Group, Holmdel, New Jersey, United States

## Abstract

Little is known about vaccination rates in home health and hospice populations. Results draw upon two separate data sources from The Visiting Nurse Association Health Group (VNAHG). Among VNAHG patients surveyed between February 2 and March 1, 202, 24% had received at least one COVID-19 vaccine. Among vaccinated patients, roughly one quarter did not travel to get the vaccine (received inpatient vaccination). They mostly traveled by car (88%), and 70% received help from a family member. Of patients who had not received a vaccine (76%), 81% were pursuing or planning to pursue obtaining a vaccine. Additionally, of those not pursuing a vaccine, 30% indicated it was because they could not get to a vaccine site. 44% of patients in the VNAHG “in home” vaccination pilot were bedbound, and 100% of patients had ambulation difficulties that make it impossible for them to leave home. All (100%) had a health care provider(s) recommended they get the vaccine. Only 38% have internet access. A quarter tried to call to schedule a vaccine, but only one was able to speak to someone. 40% of the patients attempted to get a COVID-19 vaccine prior to enrollment in the program. Most patients (81%) did not have someone available to assist with their transportation to get vaccinated, and most indicated difficulty securing an appointment. Many indicated severe traveling difficulties (requiring oxygen, needing ambulance transport). These findings highlight the high barriers for homebound patients, and the need and value of clinicians traveling to provide in-home vaccines.

